# Subcutaneous Defibrillation and Coronary Sinus Pacing After Ventricular Fibrillation With Right Ventricular Metastasis

**DOI:** 10.1016/j.jaccas.2026.108064

**Published:** 2026-04-30

**Authors:** Mert Tokcan, Amr Abdin, Christian Werner, Alhasan Almasri, Alessandro Lehr, Saarraaken Kulenthiran, Günther Schneider, Ingrid Kindermann, Andreas Link, Thorsten Kessler

**Affiliations:** a Klinik für Innere Medizin III-Kardiologie, Angiologie und Internistische Intensivmedizin, Saarland University Medical Center and Saarland University, Homburg, Germany; b HOMICAREM (HOMburg Institute of CArdioREnalMetabolic Medicine), Medical Faculty, Saarland University, Germany; c Klinik für Diagnostische und Interventionelle Radiologie, Saarland University Medical Center and Saarland University, Homburg, Germany

**Keywords:** cardiac metastasis, coronary sinus pacing, renal cell carcinoma, subcutaneous implantable cardioverter-defibrillator, ventricular fibrillation

## Abstract

**Background:**

Cardiac metastases can trigger malignant ventricular arrhythmias and limit transvenous therapy with right ventricular (RV) involvement.

**Case Summary:**

A 67-year-old patient with metastatic clear-cell renal cell carcinoma and RV metastasis developed ventricular fibrillation during ambulance transport after chest pain and a hypertensive crisis. Coronary angiography revealed severe 3-vessel coronary artery disease, followed by high-risk percutaneous coronary intervention. For secondary prevention, a subcutaneous implantable cardioverter-defibrillator (S-ICD) was implanted because RV lead placement was considered unsafe. Subsequent syncope due to sinus arrest led to discontinuation of beta-blocker therapy and was followed by recurrent ventricular tachycardia treated with 6 appropriate S-ICD shocks. Implantation of a dual-chamber pacemaker with a coronary sinus ventricular lead enabled resumption of beta-blocker therapy, with no further events before discharge.

**Discussion:**

In RV tumor involvement where transvenous leads may be unsafe, this case highlights a hybrid strategy providing defibrillation and bradycardia support.

**Take-Home Message:**

S-ICD plus coronary sinus ventricular pacing is a practical option when transvenous RV lead placement is not feasible.

## Visual Summary

**Figure:**
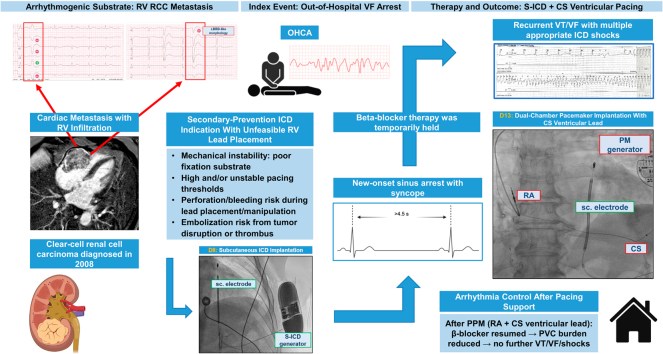

## History of Presentation

**Figure 1:**
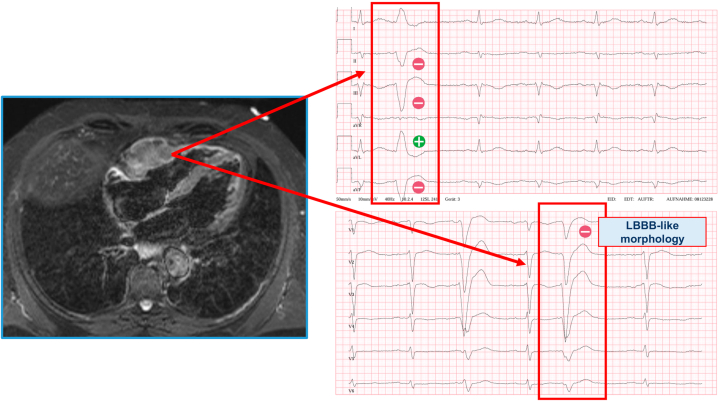
Right Ventricular Metastasis and LBBB-Like Ventricular Ectopy Cardiac magnetic resonance image (left) demonstrates a right ventricular mass consistent with myocardial infiltration by metastasis. Twelve-lead electrocardiograms (right) show frequent premature ventricular complexes and a broad-complex ventricular tachycardia pattern with LBBB-like morphology, supporting a ventricular origin and an arrhythmogenic substrate related to the right ventricular lesion. LBBB = left bundle branch block.

A 67-year-old patient required emergency services for retrosternal chest pain and hypertensive crisis. The patient was initially awake and responsive. The prehospital electrocardiogram showed no ST-segment elevation but demonstrated a high burden of premature ventricular complexes (PVCs). PVC morphology was consistent with a right ventricular (RV) origin, concordant with the known RV metastasis location (Figure 1).

**Take-Home Messages**

• When RV lead placement is precluded by intramyocardial tumor, pairing an S-ICD with a pacemaker using a coronary sinus ventricular lead can provide both defibrillation protection and bradycardia support.
• This strategy may enable guideline-concordant antiarrhythmic pharmacotherapy when bradycardia otherwise limits treatment.

During ambulance transport, ventricular fibrillation (VF) occurred, requiring brief cardiopulmonary resuscitation and defibrillation, with return of spontaneous circulation. On arrival at the emergency department, the patient was hemodynamically stable and awake. Initial laboratory testing revealed severe hypokalemia (∼2.5 mmol/L) and a mild elevation in high-sensitivity troponin T (∼60 pg/mL).

The initial clinical question was whether VF was driven by acute coronary syndrome, electrolyte disturbance, tumor-related structural substrate, or a combination of these factors.

## Past Medical History

**Figure 2:**
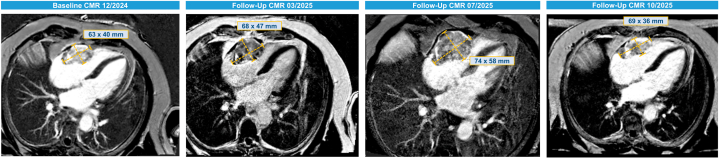
Serial CMR Follow-Up Showing Evolution of Right Ventricular Metastasis Size Serial cardiac magnetic resonance images at baseline and follow-up time points demonstrate the right ventricular mass in comparable views. CMR = cardiac magnetic resonance.

The patient was diagnosed with metastatic clear-cell renal cell carcinoma (RCC) in 2008 and subsequently underwent multiple metastasectomies, including pancreatic, pulmonary, lymph node, and adrenal resections. Systemic therapy included pembrolizumab plus axitinib (36 administrations) followed by cabozantinib from mid-2025 onward. Cardiac magnetic resonance had demonstrated a large intramyocardial RV metastasis, with interval progression by mid-2025 and subsequent regression in size and enhancement by October 2025 while on cabozantinib (Figure 2, Video 1, Video 2, Video 3, Video 4). Comorbidities included arterial hypertension and type 2 diabetes mellitus.

## Differential Diagnosis

In this patient with out-of-hospital VF arrest without ST-segment elevation, the principal differential diagnoses included acute coronary syndrome/myocardial ischemia due to severe coronary artery disease, electrolyte-triggered ventricular arrhythmia in the setting of profound hypokalemia, and a structural arrhythmogenic substrate related to the intramyocardial RV metastasis.

## Investigations

Severe hypokalemia (2.5 mmol/L) was identified and corrected. In addition, the admission electrocardiogram showed frequent PVCs. The morphology suggested a RV focus in the region of the known metastasis. Cardiac biomarkers demonstrated a modest dynamic change with high-sensitivity troponin T rising to approximately 120 pg/mL on the following day. Coronary angiography revealed severe 3-vessel coronary artery disease. Cardiac imaging documented a large intramyocardial RV mass consistent with metastatic RCC, with heterogeneous enhancement on CMR and no hemodynamically relevant RV outflow tract obstruction on echocardiography.

## Management

**Figure 3:**
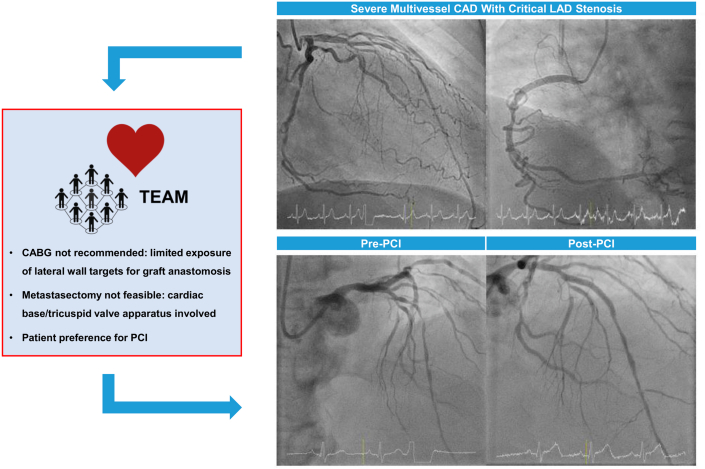
Multidisciplinary Decision-Making and Coronary Angiography Before and After Percutaneous Coronary Intervention Heart team discussion summary (left) outlining revascularization strategy in the context of severe multivessel coronary artery disease with critical left anterior descending artery stenosis. CABG = coronary artery bypass grafting; CAD = coronary artery disease; LAD = left anterior descending artery; PCI = percutaneous coronary intervention.

Immediate management focused on correcting hypokalemia and identification and treatment of reversible etiologies. Given the pronounced PVC burden, beta-blocker therapy was uptitrated to reduce ectopy and presumed triggered activity. In light of the chest pain, troponin dynamics, and VF arrest, the patient underwent an initial diagnostic coronary angiogram. After heart team review, a second procedure was performed using a high-risk percutaneous coronary intervention strategy, including drug-eluting stent implantation to the left anterior descending artery and adjunctive treatment of the diagonal branch with lesion preparation and drug-coated balloon therapy (Figure 3, Videos 5 and 6). To address the serially calcified stenoses of the mid-left circumflex artery, a staged percutaneous coronary intervention was planned.

During multidisciplinary heart team discussion, surgical revascularization was not recommended because the lateral wall was considered poorly suitable for bypass graft anastomosis, and concomitant surgical resection of the RV metastasis was deemed technically infeasible due to involvement of the cardiac base and the tricuspid valve apparatus. Accordingly, an interventional strategy was pursued in line with patient preference.

**Figure 4:**
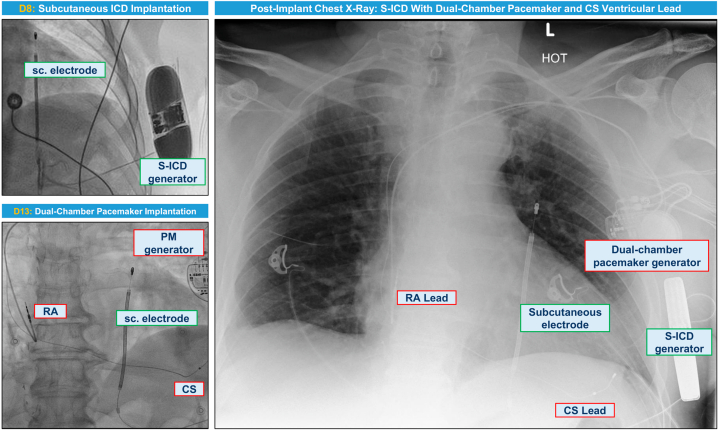
Device Therapy Combining S-ICD and Transvenous Atrial Plus Coronary Sinus Ventricular Pacing Procedural fluoroscopy and postimplant chest radiograph documenting the combined device configuration: S-ICD implanted on hospital day 8, followed by dual-chamber pacemaker implantation with a coronary sinus ventricular lead on hospital day 13 after onset of sinus arrest. CS = coronary sinus; ICD = implantable cardioverter-defibrillator; PM = pacemaker; RA = right atrium; sc. = subcutaneous; S-ICD = subcutaneous implantable cardioverter-defibrillator.

Because the RV was extensively involved by an intramyocardial tumor mass, transvenous RV lead implantation was considered unsafe and potentially unreliable. Concerns included poor lead fixation due to mechanical instability, an increased risk of perforation and bleeding during lead manipulation, potential embolization from tumor disruption, and elevated and/or unstable pacing thresholds in infiltrated myocardium—particularly in the context of a dynamic metastatic process with treatment-related changes that could further alter capture thresholds over time. Given stable oncologic disease and anticipated survival >1 year, secondary prevention defibrillator therapy was pursued in accordance with patient preference, and a subcutaneous implantable cardioverter-defibrillator (S-ICD) was implanted (Figure 4). In anticipation of a possible future cardiac surgery, the subcutaneous electrode was positioned 1 fingerbreadth left parasternal to facilitate potential surgical access to the heart, should it be required in the future. The PRAETORIAN score, which estimates the risk of ineffective S-ICD therapy based on generator position and lead course on biplane chest radiographs, was <90, indicating a >99% chance of successful defibrillation.^1^ Therefore, defibrillation threshold testing was not performed. Shock impedance after a manual 10-J test shock was normal (55 Ω) and overall system impedance was also within the normal range (65 Ω).

**Figure 5:**
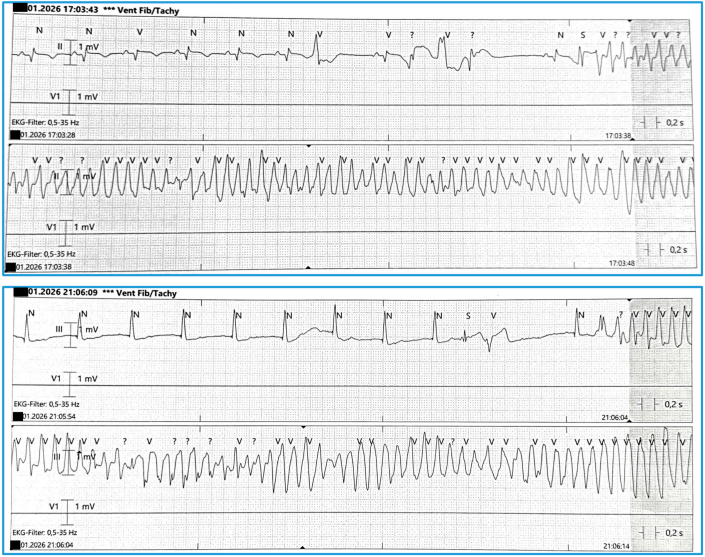
Recurrent Ventricular Tachycardia Episodes Rhythm strips showing ventricular tachycardia episodes with initiation from preceding beats and progression to sustained tachyarrhythmia consistent with recurrent ventricular tachycardia/ventricular fibrillation requiring appropriate device therapy.

A few days after S-ICD implantation, and in the absence of any previously documented bradyarrhythmias or sick sinus syndrome, the patient experienced syncope with head trauma. Holter monitoring demonstrated a sinus arrest lasting approximately 5 seconds, leading to temporary discontinuation of beta-blocker therapy. Shortly thereafter, recurrent polymorphic ventricular tachycardia (PVT)/Torsades de Pointes occurred and was treated by the S-ICD. Review of stored electrograms indicated that episodes were initiated by short-coupled PVCs (Figure 5). Device interrogation documented 6 appropriate shocks and additional untreated PVT episodes.

To permit antiarrhythmic pharmacotherapy while avoiding RV lead placement, a dual-chamber pacemaker was implanted with the ventricular lead placed in the coronary sinus (Figure 4). Pacing polarity was programmed bipolar/bipolar as recommended, and the S-ICD was programmed to primary sensing vector without amplification to reduce the risk of S-ICD oversensing. A detailed list of the devices, models, and equipment used is provided in Supplemental Table 1.

## Outcome and Follow-Up

At follow-up interrogation, the S-ICD demonstrated normal function and confirmed appropriate therapy delivery during the period of electrical storm. Pacemaker interrogation showed stable parameters, no stored arrhythmia episodes, predominant atrial pacing (∼97%), and a low ventricular pacing burden (∼4.6%) with preserved intrinsic atrioventricular conduction. After resumption of beta-blocker therapy enabled by pacing support and concomitant reduction in PVC burden, no further PVT occurred before discharge. The patient continued oncologic therapy with cabozantinib and remained on guideline-directed secondary prevention measures after percutaneous coronary intervention (Table 1).

**Table 1:** Clinical Timeline and Key Management Steps

Date /Hospital Day	Events
Day 1	Patient presents with retrosternal chest pressure and hypertensive crisis at home; initially awake and responsive. During EMS transport: ventricular fibrillation → CPR/defibrillation → ROSC.
Day 1 (ED/ICU)	Initial evaluation: severe hypokalemia; mild troponin elevation; high PVC burden on ECG with morphology consistent with RV origin matching known RV metastatic region. Electrolytes corrected; rhythm monitoring initiated.
Day 1-2 (cath 1)	Diagnostic coronary angiography shows severe 3-vessel CAD.
Day 2 (heart team)	Multidisciplinary heart team: surgical revascularization not recommended (limited graftability); surgical metastasis resection not feasible (tumor location/extent including proximity to valve apparatus/cardiac base). Plan for interventional strategy.
Day 5 (cath 2)	High-risk PCI of LAD/diagonal system (DES to LAD; adjunct lesion preparation and DCB strategy in diagonal branch) with angiographically good result. Guideline-directed secondary prevention initiated/optimized.
Day 8	Given secondary prevention indication and RV lead not feasible due to intramyocardial RV metastasis, S-ICD implantation performed. Postimplant monitoring shows no evidence of clinically relevant bradycardia/sinus pauses over subsequent days.
Day 11	Patient develops syncope; telemetry/Holter documents sinus arrest (∼5 s). Beta-blocker held due to bradyarrhythmia.
Day 11-12	Electrical storm: recurrent VT initiated by short-coupled PVCs; S-ICD interrogation shows multiple episodes including 6 appropriate shocks and additional untreated VT episodes.
Day 13	To enable antiarrhythmic therapy while avoiding RV instrumentation: dual-chamber pacemaker implanted with RA lead + coronary sinus ventricular lead. Programming adjusted to minimize S-ICD oversensing risk (bipolar pacing). Beta-blocker resumed/uptitrated to reduce PVC burden.
Day 14	Device follow-up: stable parameters; no further VT; atrial pacing predominance with low ventricular pacing burden; patient clinically stable for discharge planning.
Day 16	Discharged home in stable condition.

CAD = coronary artery disease; CPR = cardiopulmonary resuscitation; DCB = drug-coated balloon; DES = drug-eluting stent; ECG = electrocardiogram; ED = emergency department; EMS = emergency medical services; ICU = intensive care unit; LAD = left anterior descending artery; PCI = percutaneous coronary intervention; PVC = premature ventricular complex; RA = right atrium; ROSC = return of spontaneous circulation; RV = right ventricular; S-ICD = subcutaneous implantable cardioverter-defibrillator; VT = ventricular tachycardia.

## Discussion

Cardiac involvement from RCC most commonly involves the heart via contiguous tumor thrombus extension through the renal vein and inferior vena cava into the right atrium.^2^ Clinically manifest cardiac metastasis without caval or right atrial involvement is rare, with only a few reported cases of RV myocardial metastasis.^3^

This case highlights the intersection of malignant structural heart disease, ischemic substrate, and device-related constraints. The index VF event occurred in the setting of profound hypokalemia, severe coronary artery disease, and a large intramyocardial RV metastasis. Notably, both the admission electrocardiogram and subsequent device electrograms demonstrated frequent PVCs with morphology consistent with an RV origin corresponding to the metastatic region, supporting a PVC-triggered mechanism for recurrent PVT/VF.^4^ Secondary prevention with an implantable defibrillator is guideline-supported after VF arrest not attributable to a fully reversible cause.^5^^,^^6^ In this patient, standard transvenous systems were limited by RV tumor involvement, and an S-ICD provided defibrillation without an intracardiac lead.^7^

The subsequent course underscored an important limitation of the S-ICD, namely the absence of sustained bradycardia pacing capability. Development of clinically significant sinus arrest necessitated withdrawal of beta-blocker therapy and was followed by recurrent PVT requiring multiple appropriate shocks. When RV lead placement is not feasible, pacing via a coronary sinus ventricular lead can provide bradycardia support while avoiding the RV tumor region, thereby enabling antiarrhythmic pharmacotherapy. Use of combined device systems requires attention to device-device interactions, and bipolar pacing can minimize the risk of S-ICD oversensing of pacing artifacts.

Finally, ICD decisions in metastatic malignancy must account for prognosis and patient goals. In this patient, stable disease and anticipated survival beyond 1 year supported the secondary prevention indication. Proactive counseling regarding future shock management and potential device deactivation are important, should therapeutic goals change.

**Visual Summary:**
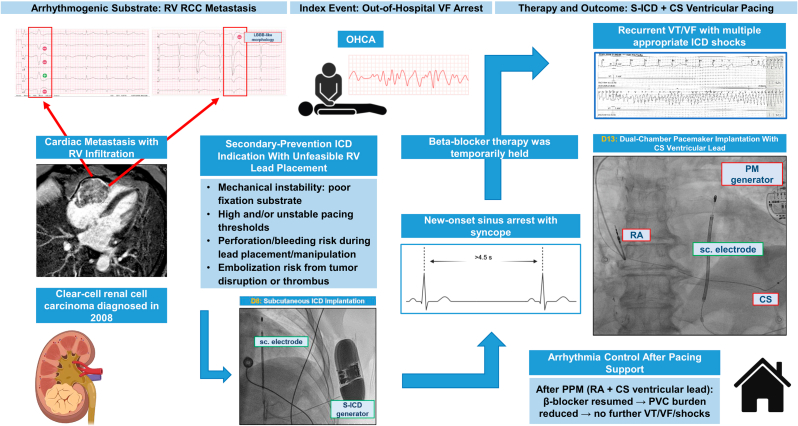
Combined Defibrillation and Pacing Strategy in Ventricular Fibrillation Arrest With RV Metastasis Overview of the clinical course in a patient with clear-cell renal cell carcinoma and cardiac metastasis infiltrating the right ventricle. Timeline from out-of-hospital cardiac arrest to subcutaneous ICD for secondary prevention, sinus arrest, and dual-chamber pacemaker implantation with coronary sinus ventricular lead. CS = coronary sinus; ICD = implantable cardioverter-defibrillator; OHCA = out-of-hospital cardiac arrest; PM = pacemaker; PPM = permanent pacemaker; PVC = premature ventricular complex; RA = right atrium; RCC = renal cell carcinoma; RV = right ventricular; sc. = subcutaneous; S-ICD = subcutaneous implantable cardioverter-defibrillator; VF = ventricular fibrillation; VT = ventricular tachycardia.

## Conclusions

In a patient with metastatic RCC and a large intramyocardial RV metastasis, VF occurred in a multifactorial context including severe hypokalemia and advanced coronary artery disease. When transvenous RV lead placement was deemed unsafe, an S-ICD provided secondary prevention defibrillation. Subsequent sinus arrest and temporary withdrawal of beta-blocker therapy precipitated recurrent PVT treated by multiple appropriate shocks, prompting implantation of a dual-chamber pacemaker with a coronary sinus ventricular lead. This combined device strategy can deliver both defibrillation protection and bradycardia support when standard transvenous RV lead placement is not feasible.

## Funding Support and Author Disclosures

Dr Tokcan received personal fees from Recor Medical and is supported by the Hans und Ria Messer Stiftung (HMS 20243118), which are unrelated to this work. Dr Kessler received personal fees from Abbott, AstraZeneca, Bristol-Myers Squibb, Recor Medical, Shockwave Medical, and Translumina, which are unrelated to this work; and is named inventor on patent applications for prevention of restenosis after angioplasty and stent implantation and for the prevention and treatment of atherosclerosis using antisense oligonucleotides, all of which are unrelated to the submitted work. Dr Werner received speaker honoraria from Amgen and Daiichi-Sankyo, unrelated to this manuscript. Dr Abdin received speaker honoraria from Boston Scientific, Medtronic, Abbott, and Bayer, unrelated to this manuscript. The authors have reported that they have no relationships relevant to the contents of this paper to disclose.

## Appendix

Video 1 Cardiac Magnetic Resonance 4-Chamber View Showing Right Ventricular Metastasis Cine steady-state free precession 4-chamber view demonstrating the intramyocardial right ventricular metastasis.

Video 2 Cardiac Magnetic Resonance Short-Axis View Showing Right Ventricular Metastasis Cine steady-state free precession short-axis view demonstrating the extent of the right ventricular metastasis.

Video 3 Cardiac Magnetic Resonance Dynamic Contrast-Enhanced 4-Chamber View Showing Right Ventricular Metastasis Dynamic contrast-enhanced 4-chamber view demonstrating contrast enhancement of the right ventricular metastasis.

Video 4 Cardiac Magnetic Resonance Dynamic Contrast-Enhanced Short-Axis View Showing Right Ventricular Metastasis Dynamic contrast-enhanced short-axis view demonstrating contrast enhancement and intramyocardial extent of the right ventricular metastasis.

Video 5 Coronary Angiography Before Coronary Intervention of the Left Anterior Descending Artery and Diagonal Branch Coronary angiography demonstrating severe disease of the left anterior descending artery and diagonal branch before revascularization.

Video 6 Coronary Angiography After Coronary Intervention of the Left Anterior Descending Artery and Diagonal Branch Final angiographic result after high-risk percutaneous coronary intervention of the left anterior descending artery and diagonal branch, including drug-eluting stent implantation to the left anterior descending artery and adjunctive lesion preparation and drug-coated balloon treatment of the diagonal branch.

Supplemental Table 1 Device and Equipment List
