# Supplementary material for: Subcutaneous Defibrillation and Coronary Sinus Pacing After Ventricular Fibrillation With Right Ventricular Metastasis
Source: JACC Case Rep. 2026 Apr 30;31(22):108064. doi: 10.1016/j.jaccas.2026.108064 (PMC13244015; doi:10.1016/j.jaccas.2026.108064)
Supplement: Supplemental Table 1 — Device and Equipment List [file mmc7.docx]

**Subcutaneous Defibrillation and Coronary Sinus Pacing After Ventricular Fibrillation With Right Ventricular Metastasis**

Short title: Hybrid Defibrillation and CS Pacing in RV Metastasis

Mert Tokcan, MD^1,2^, Amr Abdin, MD^1^, Christian Werner, MD^1^, Alhasan Almasri^1^, MD, Alessandro Lehr^1^, MD, Saarraaken Kulenthiran, MD^1,2^, Günther Schneider, MD^3^, Ingrid Kindermann, MD^1^, Andreas Link, MD, MA^1^, Thorsten Kessler, MD^1,2^

1. Klinik für Innere Medizin III – Kardiologie, Angiologie und Internistische Intensivmedizin, Saarland University Medical Center and Saarland University, Homburg, Germany
2. HOMICAREM (HOMburg Institute of CArdioREnalMetabolic Medicine), Medical Faculty, Saarland University, Germany
3. Klinik für Diagnostische und Interventionelle Radiologie, Saarland University Medical Center and Saarland University, Homburg, Germany

**Supplemental table 1:**

Equipment list3

**Supplemental table 1: Equipment List**

| **Category** | **Equipment** |
| --- | --- |
| Coronary angiography / PCI | Guide catheter: EBU 3.75 |
| Coronary angiography / PCI | Guidewire: Runthrough |
| Coronary angiography / PCI | Noncompliant balloons: 2.0×15 mm; 2.5×15 mm; NC 3.0 mm (post-dilation) |
| Coronary angiography / PCI | Drug-eluting stent: PREMIER Select 3.0×32 mm |
| Coronary angiography / PCI (LAD/Diagonal) | Scoring balloon: NCE 2.5×13 mm |
| Coronary angiography / PCI (LAD/Diagonal) | Drug-coated balloon: Prevail 2.5×30 mm |
| Subcutaneous ICD | Generator: EMBLEM S-ICD A209 (Boston Scientific) |
| Subcutaneous ICD | Electrode/lead: EMBLEM S-ICD 3501, 45 cm (Boston Scientific) |
| Pacemaker (dual-chamber) + CS ventricular pacing | Generator: Attesta DR MRI (Medtronic) |
| Pacemaker (dual-chamber) + CS ventricular pacing | RA lead: CapSureFix 5076, 52 cm (Medtronic) |
| Pacemaker (dual-chamber) + CS ventricular pacing | CS ventricular lead: Attain Ability 4196, 88 cm (Medtronic) |
